# Necrotizing soft tissue infections: a surgical narrative review

**DOI:** 10.1007/s13304-025-02222-0

**Published:** 2025-04-28

**Authors:** Silvia Tedesco, Marta Di Grezia, Giuseppe Tropeano, Gaia Altieri, Giuseppe Brisinda

**Affiliations:** 1 Emergency Surgery and Trauma Center, Department of Medical and Surgical Sciences, Fondazione Policlinico Universitario A Gemelli, IRCS, 00168 Rome, Italy; 2 Catholic School of Medicine, “Agostino Gemelli”, 00168 Rome, Italy

**Keywords:** Humans, Soft tissue infections/therapy/diagnosis, Debridement, Anti-bacterial agents/therapeutic use, Necrosis, Fasciitis, Necrotizing/therapy/diagnosis, Negative pressure wound therapy

## Abstract

Necrotizing soft tissue infections represent a spectrum of diseases characterized by extensive necrosis involving the skin, subcutaneous tissues, fascia or muscles. These infections are generally severe and rapidly progressive, often accompanied by sepsis, septic chock, multiple organ failure and, ultimately, death. Several classifications have been developed based on multiple parameters, such as the anatomical location of the disease, the depth of the lesion or the microbiology. Numerous clinical factors predispose individuals to the development of necrotizing soft tissue infections. The clinical presentation is not always characterized by local signs and systemic symptoms of infection, which can lead to delays in both diagnosis ad treatment. Broad-spectrum antibiotic directed at the likely organisms is essential early in the treatment course, but do not substitute surgical management. Antibiotic therapy should be subsequently tailored to the etiologic micro-organism. Rapid recognition and early surgical intervention form the mainstay of management of necrotizing soft tissue infections. Initial surgical debridement should be promptly performed preferably at the presenting hospital, when adequate infrastructure and personnel are available. Transfer to a referral center may be necessary for definitive surgical and complex wound care. Most patients require more than one debridement. A multidisciplinary approach is also essential to improve the results in the treatment of these patients.

## Introduction

Necrotizing soft tissue infections (NSTIs) are critical illnesses characterized by rapid tissue necrosis in the soft tissues of the body [1–3]. These infections typically involve the fascia, subcutaneous tissue, and sometimes muscle, leading to significant morbidity and potential mortality if not treated promptly [4, 5].

Although, from a purely surgical standpoint, the therapeutic options—such as extensive necrosectomy, drainage of purulent collections, or fasciotomies—are technically straightforward to perform, the fundamental pitfall of this condition remains its diagnosis [6, 7]. Currently, there are no laboratory or radiological elements that provide a definitive diagnosis. Evidence suggests that, although certain scoring systems and typical clinical signs can strongly suggest the diagnosis, the only way to confirm it is through surgical intervention [8].

Identifying patients potentially affected by this condition and determining the appropriate surgical indication is a challenging task for the surgeon. The goal is to minimize the incidence of both minor and major complication, particularly mortality. In this review, we analyzed data from the literature to identify significant clinical signs and parameters that may raise strong suspicion, as well as to determine the most appropriate surgical timing for surgery to improve patient outcomes. 

### Definition, epidemiology and etiology

**Fig. 1:**
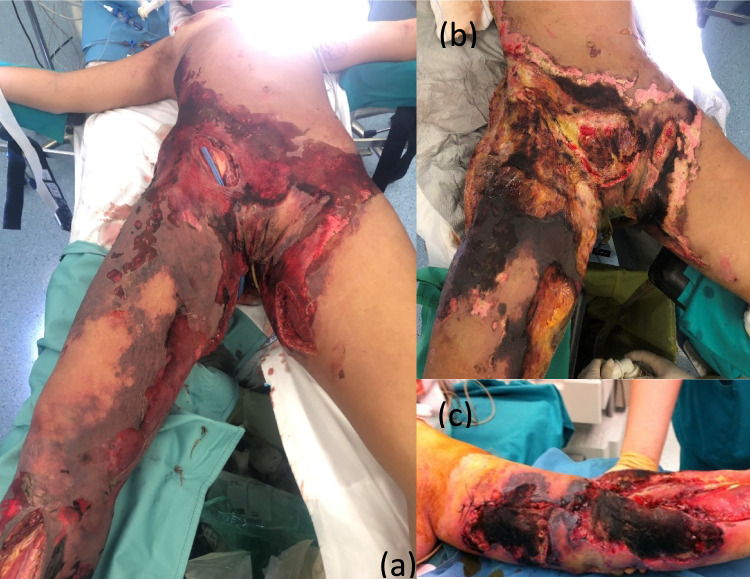
Severe NSTI. **a**-**b** Multifocal NSTIs, with involvement of trunk, perineum and lower limbs. **c** NSTIs of upper limbs. [Personal observation—Courtesy of Fondazione Policlinico Universitario A Gemelli, IRCCS, Rome, Italy]

The term NSTIs encompasses a range of infections that can occur in various anatomical locations following a breach in the integrity of the skin or mucosa. In 1869, Jones [9] provided one of the earliest descriptions about a NSTIs in a large group of patients. Over time, numerous descriptions of NSTIs have been published, often using a variety of terms that can be confusing. NSTIs can develop in any area of the body [10] but are most commonly observed in the extremities, perineum (Fig. 1) and genitalia, where they are referred to as Fournier’s gangrene [11]. Involvement of the trunk or head and neck regions is less frequent. To encompass all forms of necrotizing infections (necrotizing fasciitis, Fournier’s gangrene, synergistic gangrenes, gas gangrene, necrotizing cellulitis, myonecrosis), the term NSTIs has been proposed. However, since all NSTIs share similar pathophysiological features, distinguishing between these categories does not significantly aid surgeons in determining prompt and adequate therapy.

The Centers for Disease Control and Prevention (CDC) reported an incidence of 500 to 1500 cases yearly. More data are available regarding NSTIs caused by Group A Streptococcus (GAS) [12]. The incidence of invasive GAS infections was found to be between 2.4 and 3.1 per 100,000 person-years, with variations observed based on the time of year and the specific countries studied [13, 14].

Infection requires inoculation of the pathogen into subcutaneous tissue. In 10%–38% of patients with NSTIs, local trauma is identified as the portal of entry [15]. Such trauma can range from minor skin abrasions or insect bites to surgical injuries or blunt trauma. NSTIs may also arise in the context of chronic wounds or underlying dermatoses.

Conditions that increase the risk of these infections include an untreated perirectal abscess, any weakness in the immune system, diabetes, cancer, smoking, autoimmune diseases, and obesity. However, NSTIs can also affect young and healthy individuals with no underlying medical conditions. Co-morbidities associated with NSTIs include diabetes mellitus, present in 22 to 59% of cases, and obesity, affecting 17–31% [16]. Other notable risk factors include cardiovascular disease (9–45%), peripheral vascular disease (3–19%), intravenous drug use (2–80%), immunosuppression (4–30%), and chronic alcohol abuse (6–27%) [17].

## Classification

NSTIs can be classified based on their anatomical locations, the depth of infection (including cellulitis, adipositis, fasciitis, and myositis), and the specific microbiological organisms involved. However, these classification methods are often not clinically beneficial, as the diagnostic techniques and treatment approaches remain largely the same. 

The bacterial etiology of NSTIs is important because it can influence clinical presentation and the efficacy of certain adjunctive treatments [18]. NSTIs are typically grouped into four bacteriologic classes, a classification system originally proposed by Giuliano et al. [19]. Each class features distinct microbial causes and may reflect differences in patient demographics and typical presentations (Table 1). Nevertheless, studies have shown no significant differences in clinical outcomes, morbidity, or mortality across these groups.

**Table 1:** Microbiological classification of necrotizing soft tissue infections

Types	Etiology	Organism(s)	Characteristics	Mortality
I (70–80% of cases)	Polymicrobial/synergistic, often bowel flora-derived	Mixed anaerobes and aerobes	More indolent, better prognosis, easier to recognize. The affected anatomical locations typically include the trunk and perineum. Patients are usually older and often have multiple medical comorbidities, such as diabetes, with many lacking a clear history of trauma.	Variable, depends on underlying comorbidities
II (20–30% of cases)	Often monomicrobial, skin or respiratory-derived	Usually A β-hemolytic *streptococcus* (GAS), occasionally *S. aureus*	Aggressive presentation easily missed. Compared to Patients are generally younger, healthier, and often have a history of trauma, surgery, or intravenous drug use.	> 30%, depends on associated myositis
III (more common in Asia)	Gram-negative, often marine-related organisms	*Vibrio* spp.	Seafood ingestion or water contamination in wounds. Clinically, the infections present with early signs of significant systemic toxicity, multisystem organ failure and cardiovascular collapse can occur rapidly, sometimes without any localized cutaneous evidence of infection.	30–40%
IV (Fungal)	Trauma associated	Candida spp., immunocompromised patients. Zygomycetes in immunocompetent patients.	Aggressive with rapid extension, especially if immunocompromised patients	> 50%, higher if immunocompromised

It is essential to understand that the various types of NSTIs are not restricted to the specific patient populations outlined in these classifications. Therefore, clinicians should base treatment decisions on the overall clinical status of the patient, rather than solely on demographic factors.

Clostridial infections, traditionally associated with gas gangrene, represent a subtype of NSTIs that holds historical significance and warrants special attention within Type I NSTIs [20]. Although these infections are relatively rare—likely due to advancements in sanitation and hygiene—they remain a concern. Various clostridial species, such as *Clostridium perfringens*, *C*. *septicum*, and *C*. *sordellii*, have been associated with NSTIs among IV drug users injecting “black tar” heroin subcutaneously. Two primary toxins are thought to be most responsible for their lethality: α-toxin and θ-toxin. Initially, toxins induce a local ischemic condition at the infection site, lowering tissue pH and creating an environment conducive to bacterial proliferation. As the infection progresses and both α- and θ-toxins are absorbed systemically, they directly impair phagocyte function, cause intravascular hemolysis, decrease endothelial cell integrity, suppress cardiac function, and markedly decrease vascular tone. Bacteremia and sepsis generally occur later in the course of the infection and are associated with poor outcomes and significant mortality [21].

## Clinical presentation

Early recognition of NSTIs is crucial for effective patient management. Due to the potential lack of initial cutaneous manifestations, infections of this type were misdiagnosed at first presentation in 71% of cases, according to a systematic review involving 1463 patients. This often leads to delays in diagnosis, antibiotic treatment, and referral for surgery [22]. Patients with NSTIs typically present with signs of severe infection, including malaise, myalgia, diarrhea and anorexia, which may precede the appearance of skin lesions.

Because of the severity of illness, underlying comorbidities, and intensive postoperative wound care, admission in intensive care unit (ICU) is frequently required [23]. Between a quarter and half of patients with NSTIs develop septic shock, and some may require mechanical ventilation. Furthermore, approximately, one-third of these patients experience acute kidney injury. Organ failures often worsen within the first 24 s of admission, with ICU stay typically ranging from 5 to 12 days [24].

A systematic literature review highlighted several key clinical findings: swelling was present in 81% of NSTIs cases, pain or tenderness in 79%, erythema in 71%, warmth in 44%, bullae in 26%, skin necrosis in 24% and crepitus in 20% [22]. Fever was observed in 40% of the patients, while hypotension was present in 21%. However, the frequency of associated organ failures varies significantly across studies, largely depending on the case mix; it can rise to as high as 90% when considering only patients admitted to the ICU [25].

### Diagnosis

Laboratory findings in patients with necrotizing soft tissue infections are typically nonspecific. Common abnormalities may include leukocytosis with a left shift, acidosis, coagulopathy, hyponatremia, and elevated inflammatory markers such as C-reactive protein and erythrocyte sedimentation rate. In addition, serum levels of creatinine, lactate, creatine kinase (CK), and aspartate aminotransferase (AST) may also be elevated. Increases in serum CK or AST concentrations suggest deep infection involving muscle or fascia, as opposed to cellulitis. Wall et al. [26] proposed a model to differentiate NSTIs from non-NSTIs using criteria such as a white blood cell count exceeding 15,400/μL or a serum sodium level below 135 mmol/L. Wong et al. [27] described the Laboratory Risk Indicator for Necrotizing Fasciitis (LRINEC); it is based on laboratory indicators including white cell count, hemoglobin, sodium, glucose, creatinine, and C-reactive protein (Table 2). The LRINEC score has a sensitivity ranged from 36 to 77% and a specificity ranged from 72 to 93%. While its good specificity allows it to help differentiate NSTIs from other nonnecrotizing infections, it low sensitivity and weak positive predictive value make it a sub-optimal screening tool, insufficient for confidently identifying early NSTIs. A recent prospective, multicenter trial introduced a new clinical risk index score called necrotizing soft tissue infections (NECROSIS) aimed at identifying NSTIs in emergency general surgery patients with skin and soft tissue infections [28]. Through a comprehensive analysis of patient demographics, admission vitals, laboratory results, physical examinations, imaging, and operative findings, the authors identified three independent predictors for NSTIs: a systolic blood pressure of 120 mmHg or less, violaceous skin, and a white blood cell count exceeding 15,000/μL. The presence of all three predictors demonstrated 100% specificity and positive predictive value in both the derivation and validation cohorts.

**Table 2:** The laboratory risk indicator for necrotizing fascitis score

Variable	Value	Score
C-Reactive protein (mg/L)	≤ 150	0
	> 150	4
Total white blood cell count (1000 cells/µL)	< 15	0
	15–25	1
	> 25	2
Hemoglobin (g/dL)	> 13.5	0
	11–13.5	1
	< 11	2
Sodium (mmol/L)	≥ 135	0
	< 135	2
Creatinine (mg/dL)	≤ 1.6	0
	> 1.6	2
Glucose (mg/dL)	≤ 180	0
	> 180	1

LRINEC cutoff ≥ 6 was established for necrotizing fasciitis (NF) and patients were then classified into 3 risk groups based on their point score: low risk (< 6 points), with a < 50% risk for NF; intermediate risk (6–7 points) with 50–75% risk for NF; high risk (≥ 8 points) with a NF’s risk major of 75%

Blood cultures are positive in approximately 60 % of patients with type II NSTIs (eg, due to GAS or other β-hemolytic streptococci) and are routinely positive in patients with necrotizing myositis [29]. However, blood culture results may not reflect all organisms involved. Biomarker-based diagnostics are promising diagnostic adjuncts. Thrombomodulin has been shown to differentiate true NSTIs from suspected NSTIs. Similarly, Rath et al. [30] identified nine biomarkers that effectively differentiate β-hemolytic streptococcal NSTIs from cellulitis: IL-1β, TNF-α, CXCL8, MMP-8, IL-6, pentraxin-3, IL-22, CCL4, and S100 A8. Metabolomic analyses may also aid in the diagnosis and management of NSTIs.

Various imaging modalities—such as plain radiographs, ultrasound, computed tomography (CT), and magnetic resonance imaging—have been considered to aid in diagnosing NSTIs. However, these methods often lack sufficient sensitivity [31]. Due to concerns that imaging might delay necessary surgical intervention, current guidelines do not provide definitive recommendations for their use [32, 33]. Plain radiographs can detect gas in soft tissues, a finding that is highly specific (94%) but not sensitive (49%) for NSTIs, as gas is found in less than 25% of images [34]. Despite their limited sensitivity, plain radiographs are commonly utilized as an initial imaging step due to their accessibility [35]. CT-scans provide more detailed information compared to plain films. They can identify air in soft tissues and other subtle indicators, such as fascial enhancement or edema, with reported diagnostic sensitivity of 89% and specificity of 93% [36]. Therefore, while CT can raise suspicion of NSTIs, it should only be used when immediate surgical exploration is not required. Due to potential delays in intervention, magnetic resonance imaging is not recommended for patients with a high suspicion of NSTIs. On the other hand, ultrasound can be beneficial as it can be conducted at the bedside. A systematic review by Marks et al. [37] found that fluid accumulation along the fascial plane was the most sensitive indicator, with a sensitivity of 85.4% (95% CI, 72.2%–93.9%), while subcutaneous emphysema was the most specific, with a specificity of 100% (95% CI, 92.5%–100%).

Indocyanine green perfusion imaging may distinguish NSTIs by showing diminished fluorescence in affected tissues. Thermal infrared cameras may be used to detect temperature differentials in necrotic vs viable tissue. In a study of perineal NSTIs, preoperative infrared photos correlated with resection margins in 13 of 16 patients (81%) [38]. Further studies are necessary to determine if such modalities will affect the extent of debridement and clinical outcomes.

## Current treatment for NSTIs

**Fig. 2:**
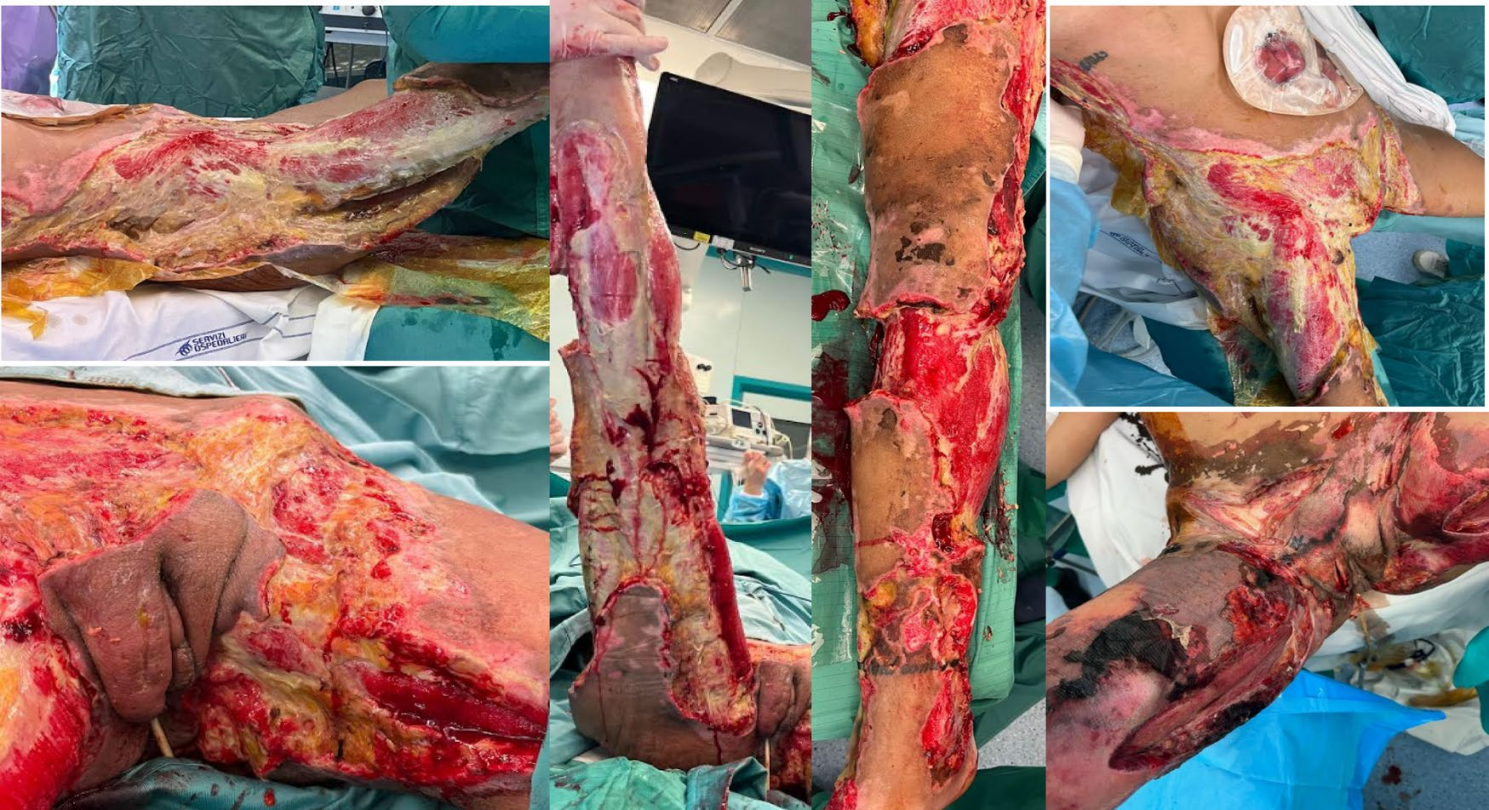
Multifocal NSTIs from Group A S. Pyogenes (GAS). Extensive surgical debridement. [Personal observation—Courtesy of Fondazione Policlinico Universitario A Gemelli, IRCCS, Rome, Italy]

Early surgical intervention is crucial for achieving complete debridement of necrotic and infected tissues (Fig. 2). Given the challenges and urgency in diagnosing NSTIs, some clinicians recommend operative exploration even when the diagnosis remains uncertain. Characteristic findings during exploration may include gray, necrotic fascia, obliterated soft tissue planes, dishwater-like fluid, absence of bleeding, thrombosed vessels, a lack of resistance to finger dissection in normally adherent tissue or noncontracting muscle. In a retrospective study involving 295 patients with suspected NSTIs who underwent surgery, Howell et al. [39] reported a 20% negative exploration rate.

For patients exhibiting signs of sepsis or septic shock, immediate surgical exploration is essential. This procedure allows for confirmation of the NSTIs diagnosis, evaluation of the need for debridement or amputation, and collection of tissue specimens for microbiological analysis. Direct examination and Gram staining of the tissue can significantly assist in diagnosing the condition and informing appropriate antimicrobial treatment.

Due to the relatively low incidence of NSTIs, many physicians and surgeons may infrequently encounter these cases. As a result, their experience in diagnosing and managing NSTIs, particularly in performing surgical debridement, may be limited. In such situations, transferring the patient to a specialized center with more expertise in NSTIs management may be advisable [8]. The decision to transfer a patient to a specialized center should be based on the experience and availability of a surgeon, the extent and severity of the skin lesions as well as the delay that would be incurred by the transfer. Often, the first debridement is done in the admitting hospital, with the patient transferred to a specialized center afterwards. Given the complexity of multidisciplinary care required for NSTIs patients, transferring them to specialized, high-volume regional centers or burn units may be beneficial [40]. These centers have been linked to lower mortality rates, likely due to their enhanced resources and expertise in managing these critical infections. However, the urgency of surgical intervention must be weighed against the desire to transfer patients [41]. Research indicates that those who are transferred may experience significantly higher mortality rates—potentially up to double—compared to patients who receive immediate treatment at the presenting hospital [40]. Therefore, initial debridement should ideally take place at the first hospital, or rapid transfer systems should be established, like protocols for other surgical emergencies [42]. One effective approach is the hub-and-spoke model, which centralizes specialized care at a hub hospital while enabling spoke hospitals to quickly transfer patients [43]. This model has been shown to enhance the proportion of patients who receive debridement within the recommended time frame [44].

## Surgical strategies

Source control through early and aggressive surgical debridement is the most important determinant of outcome in NSTIs. Current guidelines emphasize the importance of early surgical debridement in managing NSTIs. This involves excising all necrotic and infected tissue until healthy tissue is visible. Prompt intervention is crucial to prevent the spread of infection and improve patient outcomes. Since infection can extend beyond visible skin changes, it is often necessary to extend debridement beyond the initially affected areas to ensure complete removal of infected tissue. This comprehensive approach helps to prevent recurrence and promotes better healing outcomes. Culture and Gram stain of deep tissue should be obtained during operative debridement, including fungal cultures, to inform subsequent antibiotic therapy.

Kobayashi et al. [45] found that delays in surgical intervention, specifically those exceeding 12 h, are associated with a greater number of surgical debridement and an increased incidence of septic shock. This underscores the importance of timely surgical intervention in managing NSTIs to improve patient outcomes and reduce complications. Consistent with these findings, the Eastern Association for the Surgery of Trauma guidelines advocate for debridement within 12 h of suspected diagnosis [46]. An evaluation of six studies comparing outcomes between patients who underwent early (within 12 h) operative debridement versus those who did not show a significant reduction in mortality rates, with early debridement associated with 14% mortality compared to 26% for delayed interventions (*P* = 0.005) [46]. This highlights the critical importance of prompt surgical action in improving survival outcomes for patients with NSTIs. More recent joint guidelines from the World Society of Emergency Surgery, Global Alliance for Infections in Surgery, Surgical Infection Society Europe, World Surgical Infection Society and American Association for the Surgery of Trauma advocate for even earlier intervention, within 6 h of admission, citing a recent meta-analysis demonstrating that surgery within this time frame offers additional mortality benefit compared with surgery after 6 h (odds ratio, 0.43; 95% CI, 0.26–0.70; 10 studies included) [5]. The authors observed a 19% mortality rate when performed within 6 h vs 32% when delayed more than 6 h. Boyer et al. [47] demonstrated that a delay of more than 14 h from diagnosis to surgical treatment was independently associated with significantly increased hospital mortality, with an adjusted odds ratio of 34.5 (95% CI 2.05–572, *P* = 0.007). This further underscores the critical need for rapid intervention in the management of NSTIs to improve patient outcomes. In summary, when there is a high suspicion for NSTIs, surgical intervention should be initiated as soon as possible, ideally within 6 h of diagnosis.

Current guidelines recommend re-exploration every 12–24 h post-initial surgery, or sooner if there are signs of disease progression or concerning laboratory results. It is not uncommon for patients to require multiple operations—typically 3 to 4—to achieve effective source control. In a prospective observational study, Okoye et al. [48] found that repeat debridement at 24 h, compared with 48 h after initial source control, was associated with lower mortality (7% vs 33%) and less acute kidney injury (9% vs 33%).

Skin-sparing debridement has been proposed as a less morbid alternative to en bloc resection. The approach may be suitable for cases involving only fascia, when overlying skin and subcutaneous tissue are normal. In this technique, a series of counter incisions permit the removal of subcutaneous necrotic tissue while preserving viable overlying skin. The goal of a skin-sparing approach is to limit large open wounds that require skin grafting. Several authors suggest a skin-sparing approach provides equivalent source control and similar mortality rates [49]. However, given the limited data available, the decision to employ skin-sparing debridement should be approached with caution. There is a risk that inadequate debridement could result in disease progression [50]. In cases of NSTIs affecting the extremities, amputation may be necessary in up to 25% of patients, underscoring the importance of thorough surgical intervention to prevent further complications. Amputation may be considered when source control cannot be achieved with debridement, reconstruction is unfeasible or functional status is expected to be better with amputation than reconstruction [51].

At some medical centers, the anatomical location of NSTIs can influence the surgical specialty involved in treatment; for instance, Fournier’s gangrene is often primarily managed by urologists. However, due to the urgent nature of initial debridement, the availability of a surgeon may be more critical than their specific specialty. In cases of Fournier’s gangrene, a temporary diverting colostomy can be beneficial. This procedure helps to reduce fecal contamination and better manage infection in large perianal wounds. By diverting stool away from the affected area, the colostomy aids in promoting healing and minimizing the risk of further complications related to fecal exposure.

## Antibiotic therapy

While antibiotics are essential for preventing and treating systemic infection, their effectiveness is often compromised by tissue hypoxia and necrosis. Studies suggest that antibiotic therapy should be considered as an adjunct to surgical treatment, which is vital for managing patients with NSTIs. Currently, there are no randomized clinical trials specifically addressing empirical antimicrobial therapy for NSTIs, and information regarding the optimal antibiotic treatment for this condition is limited. Current guidelines and recommendations provide practical approaches based on observational studies, clinical experiences and experimental data [52]. The clinical presentation of NSTIs can vary significantly, but sepsis and septic shock are common occurrences. Therefore, it is crucial to start broad-spectrum, bactericidal antimicrobial therapy immediately upon diagnosis [53]. Given the severity of the illness and the challenges in quickly distinguishing between mixed infections and those caused by GAS, a broad-spectrum antibiotic regimen is recommended (Table 3). Empirical antifungal therapy is recommended only in immunocompromised patients. Once the specific etiology is identified and susceptibility testing is completed, the antibiotic regimen should be appropriately tailored. The duration of antimicrobial therapy for NSTIs is not clearly defined in guidelines but is generally continued until operative debridement is complete and the patient shows signs of recovery. There is no consensus on whether a fixed duration of antibiotic therapy is superior to a variable one based on clinical improvement or laboratory values [54]. Recent studies have indicated no significant differences in mortality or amputation rates between shorter durations (less than 7 days) and longer durations (more than 7 days) of antibiotic treatment [55]. The Infectious Diseases Society of America (IDSA) recommends continuing antibiotic therapy until source control is achieved and there is no further indication of systemic infection [56].

**Table 3:** Antibiotic therapy for NSTIs

Clinical conditions	Broad-spectrum therapy	Dosage
Stable patient with no shock	Amoxicillin/clavulanate*	1.2–2.2 g every 8 h
	Ceftriaxone plus Metronidazole*	2 g every 24 h 500 mg every 8 h
	Cefotaxime plus Metronidazole*	2 g every 8 h 500 mg every 8 h
Unstable patients with septic shock	Piperacillin/Tazobactam**	4.5 g every 6 h
	Meropenem**	1 g every 8 h
	Imipenem/Cilastatin**	500 mg every 6 h
	Vancomycin***	25–30 mg/kg loading dose, then 15–20 mg/kg every 8 h
	Daptomycin***	6–8 mg/kg every 24 h
	Telavancin***	10 mg/kg every 24 h

Association with *Clindamycin 600–900 mg every 8 h; **Linezolid (600 mg every 12 h) or Tedizolid (200 mg every 24 h); ***Clindamycin 600–900 mg every 8 h

## Other therapeutic options

Adjunctive therapies, including hyperbaric oxygen (HBO) therapy and intravenous immunoglobulin (IVIG), have also been proposed [57]. The rationale for using HBO would be to increase the tissue oxygen tension of infected hypoxic areas and prevent further extension of infection [58]. However, clinical evidence supporting HBO therapy is of low quality, relying mainly on uncontrolled observational case series [58]. The use of IVIG has been evaluated in observational studies on GAS toxic shock syndromes, yielding controversial results [59]. IVIG therapy has been used for NSTIs treatment, primarily to enhance bacterial opsonization and toxin neutralization. Current evidence regarding the impact of IVIG on clinical outcomes is conflicting. A recent meta-analysis assessing IVIG in clindamycin-treated patients with streptococcal toxic shock syndrome found that mortality decreased from 33.7% to 15.7% with IVIG therapy [59].

As genomic, proteomic, and metabolomic data continue to accumulate, the potential for personalizing care based on genetic signatures will enhance. The INFECT project, a multicenter prospective observational study, aims to deepen understanding of the pathophysiology and prognosis of NSTIs to improve diagnosis and tailor individualized treatment strategies [60].

## Role of negative pressure wound therapy

**Fig. 3:**
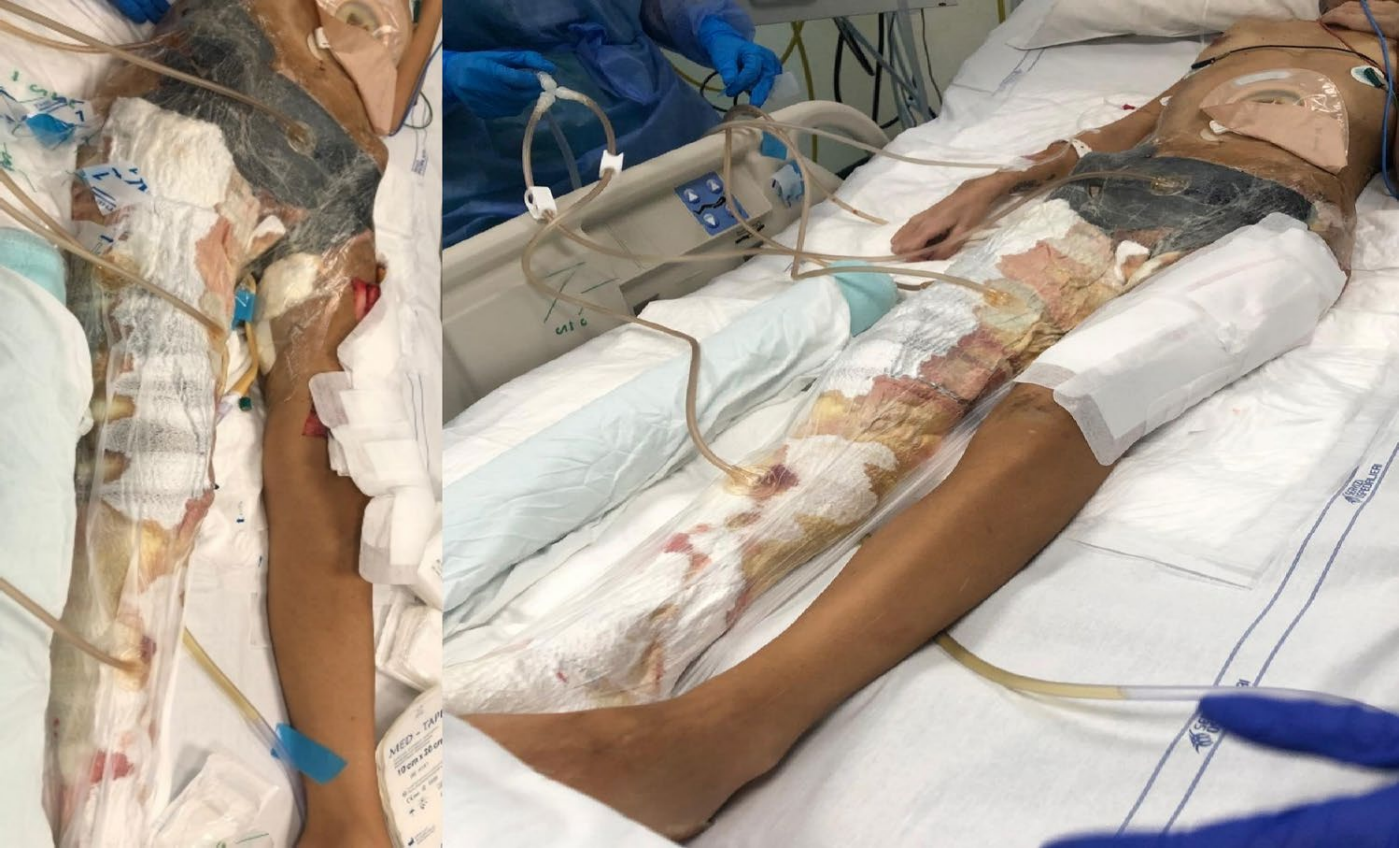
Multifocal NSTIs from Group A S. Pyogenes (GAS). NPWT application. [Personal observation—Courtesy of Fondazione Policlinico Universitario A Gemelli, IRCCS, Rome, Italy]

The goals of treatment encompass achieving a clean wound, protecting it from desiccation, assessing the need for further surgical debridement, and promoting wound healing (Fig. 3). Negative pressure wound therapy (NPWT) is an adjunctive treatment that applies sub-atmospheric pressure to the wound surface after debridement. The optimal timing for initiating NPWT and its suitability for all patients with NSTIs remain unclear. A recent metanalysis demonstrated that NPWT was associated with lower mortality but no difference in length of stay, number of debridement, or complication rates as compared with conventional dressings [61]. A recent study questioned the use of NPWT in exclusively anaerobic infections, as oxygen deprivation may be less effective against these species [62]. Given the need for frequent wound checks in the early postoperative period, it may be best to delay initiation of NPWT until the patient undergoes repeated debridement and the wound is deemed stable.

## Pain control

Patients often experience multiple painful and anxiety-inducing procedures during wound care and rehabilitation for NSTIs. To achieve optimal outcomes, it is essential to regularly evaluate and assess both pain and anxiety while administering appropriate pharmacologic and nonpharmacologic therapies. Pharmacologic therapies form the foundation of analgesia and sedation, while nonpharmacologic therapies—such as virtual reality, relaxation techniques, distraction interventions, music, massage, and hypnosis—serve as valuable adjuncts in attaining adequate pain relief. Pain can be categorized into three types: background pain, breakthrough pain, and procedural pain, each requiring separate management strategies. Patients may be discharged with a combination of narcotics tailored to address each type of pain, alongside with a comprehensive weaning protocol that accounts for their potentially higher-than-normal requirements compared to typical surgical patients. In addition, small randomized clinical studies have identified gabapentin as effective in reducing acute pain and noted its opioid-sparing effects [63].

## Wound coverage and reconstruction

**Fig. 4:**
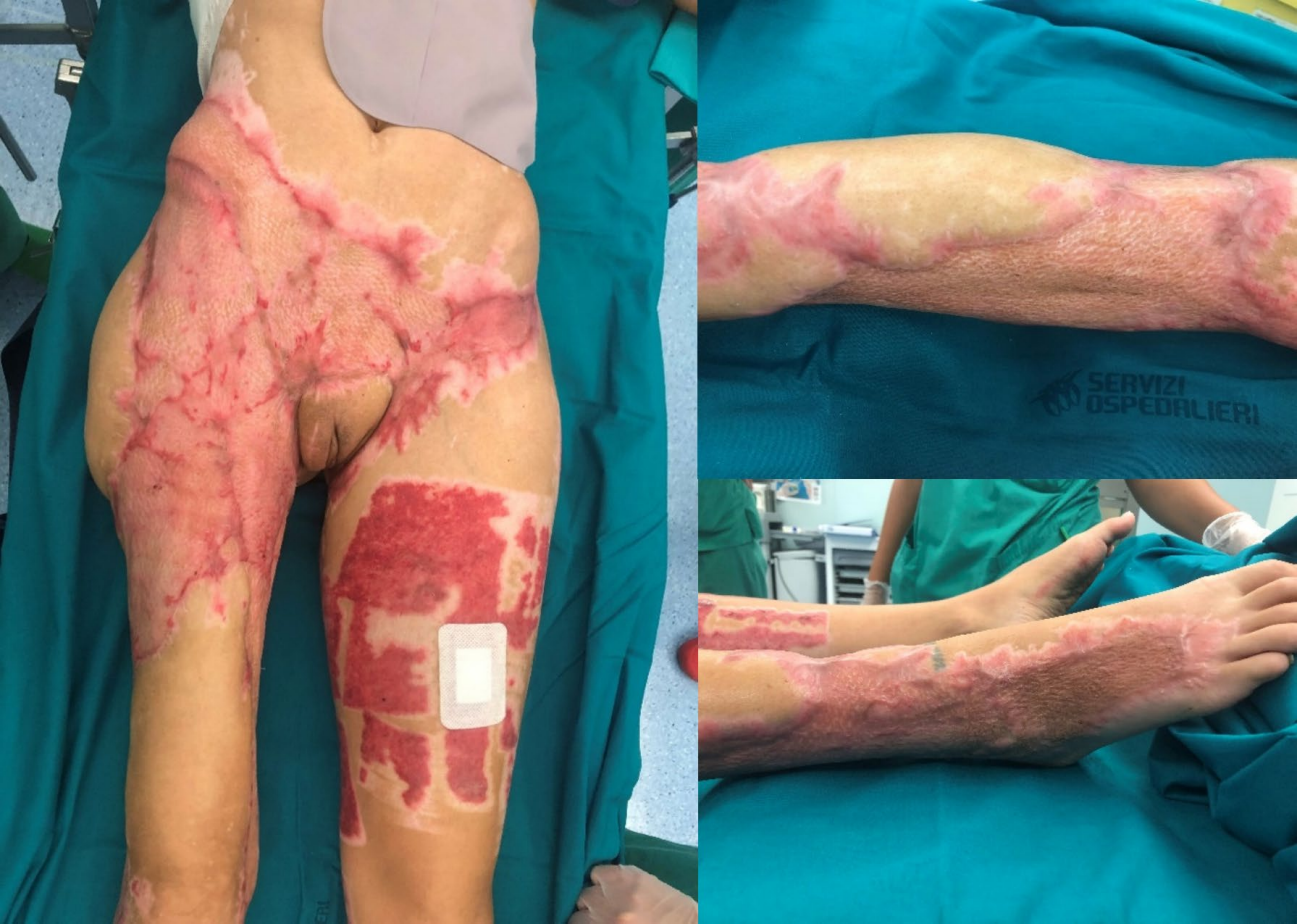
Healing process after numerous dermo-epidermal grafts, which were necessary due to extensive loss of substance in the skin and subcutaneous tissue, following multiple debridements and necrosectomies. [Personal observation—Courtesy of Fondazione Policlinico Universitario A Gemelli, IRCCS, Rome, Italy]

Traditionally, reconstructive options for NSTIs have included primary closure, split-thickness skin grafts, full-thickness skin grafts, delayed primary closure versus healing by secondary intention, tissue expansion, and pedicled or free flaps (Fig. 4). Fortunately, most NSTIs cases do not necessitate advanced techniques at the upper levels of the reconstructive ladder. In many instances, skin grafting and local tissue rearrangements are sufficient for achieving wound closure.

Temporary skin substitutes provide immediate tissue coverage, reducing fluid, protein, and electrolyte loss while offering protection from microbial invasion. They also minimize painful wound care and allow for early mobilization. This temporary coverage is especially important for devastating soft tissue defects that expose neurovascular and vital structures, which are vulnerable to desiccation and rupture. In cases where the patient is unstable, the wound is heavily contaminated, or the viability of the wound bed is uncertain, we prefer to use porcine xenografts as a temporary measure. The temporary skin substitute can remain in place until the patient stabilizes and further surgical interventions are scheduled. However, if the pigskin xenograft is left in place for more than 7–10 days, it may begin to integrate with the underlying granulation tissue, necessitating further excision to create a wound bed free of xenograft material.

AlloDerm and Integra are also utilized as dermal substitutes, playing an adjunctive role in optimizing the wound surface for eventual skin grafting [64]. AlloDerm can serve as a temporary dressing to protect exposed neurovascular structures before definitive reconstruction. It allows the underlying wound bed to granulate, and prior to final closure, it can be completely removed or used to bridge larger tissue defects. Integra is a bilayer matrix wound dressing composed of a silicone layer over a collagen-based dermal analogue.

Skin grafts are often the most convenient option but may be limited by donor site availability or concerns regarding the viability of the underlying wound bed. Selecting the right type of skin graft can also pose challenges for surgeons who are inexperienced with the procedure.

## Prognosis

Mortality is about 20–30%. Studies have shown that for each unit increase in the APACHE II score, the odds of death increase by 16% to 18% [65]. If a patient is hypotensive upon admission, the mortality rate doubles. Furthermore, the odds ratio for mortality rises to 28.4 (95% CI 1.35–77.8) if vasopressors are required at the time of ICU admission [66].

Rates of morbidity and mortality are notably higher among socioeconomically disadvantaged groups [67]. Black patients face a greater risk of complications, including a 40% higher likelihood of amputation. Meanwhile, Hispanic patients have nearly a 60% increased risk of mortality compared to their White counterparts or those with private insurance. The factors contributing to these disparities warrant further investigation.

Bacteremia upon admission has also been linked to increased mortality in patients with NSTIs. Retrospective studies have identified several factors that may suggest a poor prognosis [23]. Multiple studies have sought to identify markers indicative of disease severity and mortality risk in NSTIs. Molecules such as ICAM-1, urokinase-type plasminogen activator receptor (suPAR), pentraxin-3, and fibrocolin-2 have all shown potential value in prognosticating outcomes [68]. In addition, the use of artificial intelligence to predict the prognosis of these patients appears promising. A model utilizing 16 predictive parameters available on or before the first day of ICU care has demonstrated the ability to predict 30-day mortality more accurately than existing scoring systems [69].

Patients with NSTIs often experience significant long-term functional disabilities. Only half of the patients were able to return directly home, while the remaining individuals required additional hospitalization or transfer to an inpatient rehabilitation facility [70].

## Conclusion

NSTIs are an increasingly common problem, often characterized by progressive and fatal soft tissue infection that require prompt, radical and often multiple surgical debridement of all involved tissue. Early diagnosis of a NSTI is critical to achieve optimal outcomes. Treating physicians must be familiar with the signs and symptoms of the disease and maintain a high index of suspicion. Any delay in diagnosis is potentially catastrophic, as the concomitant delay in surgical treatment significantly increases mortality. It is evident that, in the early stages of treating a patient in the ED, the objective data available for formulating a diagnosis are extremely limited, as neither radiological nor laboratory signs have proven to be statistically significant. A multidisciplinary approach to the treatment of NSTIs is crucial, involving the surgeon, anesthesiologist, and infectious disease specialist. Surgical debridement of all necrotic tissue remains the golden standard of treatment. The massive soft tissue defect requires special attention and can present significant challenges. This may require assistance from reconstructive surgeons in concert with strong support from rehabilitative services in-hospital and post-hospital setting. 

## Data Availability

The authors confirm that the data supporting the findings of this study are available within the article. All the authors have given full approval of the version to be published.
